# Cost of illness for cholera in a high risk urban area in Bangladesh: an analysis from household perspective

**DOI:** 10.1186/1471-2334-13-518

**Published:** 2013-11-04

**Authors:** Abdur Razzaque Sarker, Ziaul Islam, Iqbal Ansary Khan, Amit Saha, Fahima Chowdhury, Ashraful Islam Khan, Firdausi Qadri, Jahangir AM Khan

**Affiliations:** 1 Enteric Vaccine, Centre for Vaccine Sciences, International Centre for Diarrhoeal Disease Research, Bangladesh (icddr,b), Dhaka, Bangladesh; 2 Health Economics and Financing, Centre for Vaccine Sciences, International Centre for Diarrhoeal Disease Research, Bangladesh (icddr,b), Dhaka, Bangladesh; 3 Health Economics Unit, Department of Learning, Informatics, Management and Ethics, Karolinska Institute, Stockholm, Sweden

**Keywords:** Cost of illness, Cholera, Urban Bangladesh

## Abstract

**Background:**

Cholera poses a substantial health burden to developing countries such as Bangladesh. In this study, the objective is to estimate the economic burden of cholera treatments incurred by households. The study was carried out in the context of a large vaccine trial in an urban area of Bangladesh.

**Methods:**

The study used a combination of prospective and retrospective incidence-based cost analyses of cholera illness per episode per household. A total of 394 confirmed cholera hospitalized cases were identified and treated in the study area during June–October 2011. Households with cholera patients were interviewed within 15 days after discharge from hospitals or clinics. To estimate the total cost of cholera illness a structured questionnaire was used, which included questions on direct medical costs, non-medical costs, and the indirect costs of patients and caregivers.

**Results:**

The average total household cost of treatment for an episode of cholera was US$30.40. Total direct and indirect costs constituted 24.6% (US$7.40) and 75.4% (US$23.00) of the average total cost, respectively. The cost for children under 5 years of age (US$21.50) was higher than that of children aged 5–14 years (US$17.50). The direct cost of treatment was similar for male and female patients, but the indirect cost was higher for males.

**Conclusion:**

Our study suggests that by preventing one cholera episode (3 days on an average), we can avert a total cost of 2,278.50 BDT (US$30.40) per household. Among medical components, medicines are the largest cost driver. No clear socioeconomic gradient emerged from our study, but limited demographic patterns were observed in the cost of illness. By preventing cholera cases, large production losses can be reduced.

## Background

Cholera presents a substantial health burden in the developing world and is endemic in Africa and Asia, and has recently spread to the Americas. An estimated 1.4 billion people worldwide are at risk of cholera; India and Bangladesh jointly constitute the largest share of this population [1]. Cholera is a waterborne disease and is closely linked to inadequate environmental management. It is responsible for 100,000–120,000 deaths per annum globally, which constitutes an estimated 3–5 million cholera cases every year [2]. However, the World Health Organization (WHO) acknowledges that only 5%–10% of cholera cases are actually reported and it is likely that their data on cholera rates are a gross underestimation of the real burden of the disease [1]. In Bangladesh, there is no accurate data on the actual number of cholera cases but estimates by experts suggest an incidence of approximately 450,000 cases each year [3,4].

Cholera cases have increased in Bangladesh, especially in urban settings such as in the capital of Dhaka, where the number of hospitalized patients with more severe cases has significantly increased [5]. Looking at diarrheal epidemics in Dhaka in 1998, 2004, and 2007, it is apparent that the number of cases of severe dehydration due to cholera is increasing with each epidemic: 22% in 1998, 25% in 2003, and 35% in 2007 [5,6]. Although cholera is one of the most prevalent diseases in the country, studies on its economic impact are limited. The Diseases of the Most Impoverished (DOMI), a cholera cost study group, carried out a multi-country cost-of-illness study in Matlab (rural Bangladesh), Beira (urban neighborhood in Mozambique), Kolkata (middle-class and slum neighborhood in India), and North-Jakarta (middle-class and slum neighborhood in Indonesia). While DOMI studied the cost of illness in the rural context in Bangladesh, it did not include any information regarding urban Bangladesh. This current study, therefore, aims to calculate the cost of illness for households due to cholera treatment in an urban area with high cholera prevalence. By addressing the information gap on the cost of illness for households in urban Bangladesh due to cholera treatment, this study offers a more complete economic perspective of the cost of cholera for health policy making in general and for prevention strategies in Bangladesh.

## Methods

### Study site and population

The “Introduction of Cholera Vaccine in Bangladesh” (ICVB) project is currently being conducted in six wards (lowest administrative units) in Mirpur (an urban area), Dhaka, Bangladesh. The Mirpur area is densely populated (approximately 2.5 million people) with a high proportion of high-risk populations prone to cholera and other diarrheal diseases. The wards in Mirpur were selected based on reports of a higher influx of diarrheal patients to Dhaka hospital of International Centre for Diarrheal Disease Research, Bangladesh (icddr,b), over the last 5 years. The estimated rate of hospitalization due to cholera is 2–6 per 1,000 people in these selected wards [5]. Patients of all ages residing in Mirpur’s six wards who were confirmed by stool culture to have *V. cholerae* O1 and hospitalized for diarrhea were eligible to participate in this study. It is worth noting that non-hospitalized patients were not included because there is no scope for cholera confirmation without laboratory testing.

### Study perspective

An incidence-based approach was applied to estimate the cost of illness of cholera treatment per episode from a household perspective. In the study, household members in the ICVB surveillance area who sought care at any health facilities and were laboratory confirmed as cholera cases by icddr,b hospitals were included. A structured questionnaire was developed to collect data on all possible cost components, including direct medical and non-medical costs as well as indirect costs incurred by the households.

### Sample size

All confirmed cholera hospitalized cases, coming from one of the six wards in Mirpur during June–October 2011, were included in the study. A total of 394 confirmed cholera cases were identified and interviewed.

### Patient enrollment

Information (name, address, cell phone number) on confirmed cholera patients was collected from health facility databases and interviews were conducted within 15 days after discharge from hospital. We interviewed the household head or the person who was most familiar with the costs incurred during the cholera treatment of the patient.

The interviews were conducted at the respondent’s residence. Written informed consent was obtained from all respondents. Structured questionnaires with both open-ended and closed questions were used [see Additional file 1] by trained data collectors to obtain data.

### Measuring household costs of cholera

Household costs of cholera episodes include out-of-pocket payments made by the households for the treatment of cholera and the opportunity costs for time used by the patients and/or caregivers during the entire cholera episode. Out-of-pocket payments consisted of direct medical and non-medical costs. Direct medical costs included hospital outpatient fees, admission or registration fees, physician fees, consultant fees, payments to paramedics during home visits for intravenous infusions, medicine costs, oral rehydrating solution, laboratory tests, diagnostic fees, and any other associated medical supplies. The direct non-medical costs include transportation, lodging, food items, tips (informal payment), payment to caregiver for loss of regular work or payment for attending patient, expenditure for materials such as utensils and other items such as mosquito coils and lighters for patients and also the cost of caregivers during the treatment.

Indirect costs were those related to income or productivity loss and were measured by applying the human capital approach. Income loss for paid workers was measured by multiplying the number of lost working hours due to a cholera episode with the actual wage rate of the patient. Self-reported wage rates have been used in this study. The productivity loss due to forgone non-market activities including school, household chores, childcare, and leisure time were captured. The value of daily productivity was measured on the basis of either an assumed age-specific wage or an occupation-specific wage as used in other studies [7]. Few studies monetized the loss associated with children [8] who have been considered to make important economic contributions to the household [9]. We assumed age-specific wages for three groups: adults, teenagers, and children aged 5 to 14 years. The average daily wages of the patients were used for adult patients, while one half and three-quarters of that wage were applied to teenagers and children, respectively. Half the average wage was assigned to unpaid home workers, taking their age group into consideration [7].

Intangible costs, i.e., costs related to suffering and grief, have been included as an additional cost category in other studies. However, such costs are not generally valued and no tangible economic impact is implied [9]. In this study, the intangible cost due to cholera was not considered.

### Data analysis

Data were entered into Microsoft Excel 2007. All entries were manually double-checked and verified by the investigators. Subsequently, statistical analysis was performed using STATA-11.1. An equivalence scale was applied to adjust for household size when calculating household income per equivalent adult [10]. Data were presented as a total and as an average with a standard deviation in local currency, i.e., Bangladeshi Taka (BDT) and US dollars (US$) applying the exchange rate (US$1 = 75 BDT) during the mid-point of the data collection year (2011).

A sensitivity analysis was conducted on direct and indirect costs to test the robustness of the assumptions and to examine the impact of potential outliers in the database [11]. Costs of informal caregivers had a higher level of uncertainty and could be different [12]. We tested the effects of a change of 20% in the parameters of both direct costs and indirect costs as performed in an earlier study [11].

### Ethical approval

The research protocol of this study was approved by the Institutional Review Board of the icddr,b.

## Results

A total of 394 patients participated in the study, of which 53% were male and 47% were female, and 36% were younger than 5 years of age. All households that were approached to participate in the survey gave written consent, thus, no household refused to partake.

The average total cost of treating one episode of cholera was found to be 2278.50 BDT (US$30.40). The direct cost was 559.50 BDT (US$7.40), which represented 24.6% of the total cost. Direct costs made up 24.6% of the average total cost, of which 8.9% and 15.6% were medical and non-medical cost components, respectively. Medicine costs made up the largest share among all direct medical cost components, followed by registration or admission fees (Table 1). Among the direct non-medical cost components, transportation constituted the largest (140 BDT or US$1.90) followed by caregiver costs (113.25 BDT or US$1.50). Food items (63 BDT or US$0.80) represented a significant proportion of direct non-medical cost as well. A wide range in cost per episode was observed in the standard deviation from the average value. For a better understanding of such a spread in cost, the median, and the 5th and 95th percentiles were calculated for each cost item. The median of the total cost was 1,306.50 BDT (US$17.40) and the distribution as 5th and 95th percentiles was 285 BDT (US$3.80) and 5,822 BDT (US$77.60), respectively. Median direct and indirect costs were 392.50 BDT (US$5.20) and 807.50 BDT (US$10.80), respectively. The 5th and 95th percentiles for direct costs were 80 BDT (US$1.10) and 1,430 BDT (US$19.10) and the corresponding values for indirect costs were 95.60 BDT (US$1.30) and 3,774 BDT (US$50.30), respectively.

**Table 1:** Average household cost of cholera treatment, BDT (US$^*^)

**Costs**	**Parameters**	**Average cost**	**Standard deviation**	**Proportion of total cost**
**Direct Medical**	Diagnostic	9.6 (0.1)	75.7 (1)	9
	Medicine	148.7 (2)	246 (3.)	
	Registration fee	26.1 (0.3)	130.6 (1.7)	
	Paramedics home visit fee	2.8 (-)	21.5 (0.3)	
	Bed/ Cabin charge	16.9 (0.2)	130.7 (1.7)	
**Direct Non-Medical**	Transportation cost	140 (1.9)	122 (1.6)	15.6
	food items	63(0.8)	85 (1.1)	
	Informal payment	0.7 (-)	9 (0.1)	
	Caregivers payment	0.1 (-)	1 (-)	
	Materials (mug/glass/coil etc.)	10.6(0.1)	17 (0.2)	
	Lodging	28 (0.4)	101 (1.3)	
	Caregivers expenditure	113.2 (1.5)	172 (2.3)	
**Total direct cost**		559.5 (7.4)	641.7 (8.5)	24.6
**In-direct**	Patients income loss	811 (11)	4,301 (57)	
	Caregivers income loss	908 (12.2)	3,701 (49)	
Total indirect cost		1,719 (23)	5,656 (75.4)	75.4
**Total cost of illness of household**		**2,278.5 (30.4)**	**5,668 (75.6)**	**100**

*1 US dollar (US$) = 75 Bangladeshi Taka (BDT) in mid 2011.

Indirect costs were 1,719 BDT (US$23) per episode per household, which represented 75.4% of the average total cost (Table 1). We also observed that the average caregiver’s production loss (908 BDT or US$12.20) was higher than that of the patient’s (811 BDT or US$11).

A one-way sensitivity analysis with a 20% increase in the parameters of direct and indirect costs showed that the total cost increased by 4.9% and 15%, respectively.

### Costs across income groups

The average total cost of the poorest (1st) quintile was 1,894.50 BDT (US$25.30) while that of the richest was 2,335.60 BDT (US$31.10) per episode. Households in the second quintile incurred the largest average total cost (2,993 BDT or US$40). No socioeconomic gradient was observed. Direct costs represented approximately 29% of total cost in the poorest, middle, and richest quintiles. The corresponding shares in the second and fourth quintiles were 15.5% and 23.3%, respectively (see Table 2).

**Table 2:** Household average cost of cholera treatment by income quintile, BDT (US$)

**Income quintile (equivalent per adult income, BDT)**	**Number of household**	**Average direct cost**	**Average indirect cost**	**Average total cost**
1 (≤1647)	82	562 (7.5)	1,332.5 (17.8)	1,894.5 (25.3)
2 (1,648-2,500)	78	474 (6.3)	2,519 (33.6)	2,993 (39.9)
3 (2,501-3,529)	80	482.9 (6.4)	1,197 (16)	1,680 (22.4)
4 (3,530 -5,333)	83	583.4 (7.8)	1,927 (25.7)	2,510.4 (33.5)
5 (5,334+)	77	696.4 (9.3)	1,639.2 (22)	2,335.6 (31.1)

### Costs across age groups

The average total costs ranged between 1,314 BDT (US$17.50) and 6,214 BDT (US$82.60). The largest cost was observed among patients aged 60 years and older. The direct costs ranged between 345 BDT (US$4.60) and 635 BDT (US$8.50) and the highest cost was observed among patients under 5 years of age. While indirect costs increased for older patients, the direct costs did not show significant disparity across age groups (see Table 3).

**Table 3:** Household average cost of cholera treatment by age group, BDT (US$)

**Age group (years)**	**Number of patients**	**Average direct cost**	**Average indirect cost**	**Average total cost**
Up to 4	131	635 (8.5)	980 (13)	1,615 (21.5)
5 to 14	34	537 (7)	778 (10)	1,314 (17.5)
15 to 45	178	529 (7)	1,733 (23)	2,261 (30)
46 to 60	39	532 (7)	3,682 (49)	4,214 (56.2)
60+	12	345 (4.6)	5,870 (78)	6,214 (82.6)

### Costs by gender

The average total costs for males and females were BDT 2,526 (US$33.80) and BDT 1,995 (US$26.50), respectively. The average direct costs were slightly more for males than females. The difference in average total cost can be explained by associated indirect costs, as a greater number of males are in the labor market. We found that the difference in indirect costs was higher for males (see Table 4).

**Table 4:** Cost of cholera treatment by sex

**Sex**	**Number of patients**	**Average cost, BDT (US$)**		
		**Direct cost**	**Indirect cost**	**Total cost**
Male	210	556 (7.5)	1,970 (26.3)	2,526 (33.8)
Female	184	563 (7.4)	1,432 (19.1)	1,995 (26.5)
Total	394	559 (7.4)	1,719 (23)	2,286 (30.4)

Among children under 5 years of age, males have a higher average total cost (1,763.40 BDT or US $23.50) than females (1,321.40 BDT or US$17.70). Both direct and indirect costs were higher for male children (see Table 5).

**Table 5:** Gender differential in average cost of cholera treatment among under-five children

**Sex**	**Number of patients**	**Average cost, BDT (US$)**		
		**Direct cost**	**Indirect cost**	**Total cost**
Male	87	642.4 (8.6)	1,121 (15)	1,763.4 (23.5)
Female	54	564.4 (7.7)	756 (10)	1,321.4 (17.7)
Total	141	612.5 (8.2)	982 (13)	1,594.2 (21.2)

## Discussion

This study found that both the average and median length of a cholera episode was 3 days and the 5th and 9th percentiles of the episode were 1 and 6 days, respectively. This costing study was carried out in the Mirpur area of Dhaka, in which vaccination trial was also carried out. In the target area, 123,661 people had received their full vaccinations. However, in our current study sample, only 84 of 394 were fully vaccinated. The data for this study were collected when the vaccine trial had just started and the effect of vaccination may not have been achieved that time.

While the cost of illness for cholera in rural Bangladesh had been investigated in an earlier study, such information was lacking for urban areas in the country. In a multi-country study, previous researchers applied hospital-based data collection techniques in rural Bangladesh to estimate the cost of illness due to endemic cholera [7]. That study also included India, Mozambique, and Indonesia. It was found that the average cost of illness for a Bangladeshi household was US$12.40. The corresponding costs in Beria (Mozambique), Kolkata (India), and North Jakarta (Indonesia) were US$18.80, US$17.90, and US$134.00, respectively [7]. The current study, however, found that the average total cost per episode of cholera illness for households is BDT 2,278.50 (US$30.40).

Medicine, transportation, caregiver costs, and opportunity costs (indirect costs) were the largest cost components in the study. Some of the costs varied across socioeconomic and demographic groups.

The total indirect cost was more than three times higher than the total direct cost. Among the direct costs, medical related costs constituted 36.4% of the total, representing the largest share. Among the non-medical components, transportation costs were the highest, followed by caregiver costs, which included food, lodging, and cell phone costs. It was found that the average total cost of illness was greater for adult patients than child patients (Table 3). The variation in average total costs across age groups can be explained by indirect costs because the direct costs do not differ greatly across groups. While the indirect costs of adult patients can be influenced by their wage level and length of cholera episode, such costs for children are also influenced by these factors (wage and length of episode) in terms of their caregiver. The data from this study show that the health-seeking behavior of adults and children differs to some extent. For instance, children are often taken to local private practitioners before hospitalization, while adult patients generally seek care directly from hospitals. A disaggregation of costs into components provides a better understanding of the cost drivers. In the two icddr,b hospitals (Dhaka and Mirpur hospitals), all diagnostic tests and medicines were provided free of charge to the patients. Although the patients received their required medicine in hospital, some patients still purchased extra medicine from nearby pharmacies. In contrast, a large amount of money was spent in private hospitals on diagnosis and medicine. In addition, private hospitals also charged registration fees that are not charged in icddr,b hospitals. In some cases, out-of-pocket payments were incurred by households (Table 1), e.g., fees for home visits from paramedics who offer various services including intravenous saline solution and providing advice.

The high costs of transportation can be explained by travel time on highly congested roads in Dhaka. The approximate travel time to private facilities was 60 minutes, whereas it took on average 80 and 48 minutes to reach the icddr,b Dhaka hospital and Mirpur hospital, respectively. It was also observed that waiting time in private hospitals was longer (16 minutes) than in the icddr,b hospitals (4.2 minutes on average). The highest waiting times (the longest was 34 minutes) were observed in private clinics and hospitals outside the cholera surveillance area (see Table 6).

**Table 6:** Reported travel and waiting time for cholera treatment

**Facility**	**Number of visit**^**1**^	**Average time spent (minute)**		
		**Travel time**	**Waiting time**	**Total time**
Local pharmacy	291	11.6	1.5	13.1
Local MBBS physicians	25	25.1	13.2	38.3
icddr,b Mirpur hospital	266	48.4	4.3	52.7
icddr,b Dhaka hospital	137	80	4	84
Private clinics (ten other health facilities)^2^	33	56	16	72
Traditional practitioner	4	12.3	0	12.3
Other private clinic^3^	10	27.3	34	61.3

^1)^Multiple visits applied; ^2)^Patients frequently visit these hospitals; ^3)^Located outside ICVB surveillance area.

This study does have some limitations. There may be some recall bias as data were collected after receiving treatment. However, to minimize the bias, we conducted all interviews within 15 days of discharge from hospital. Outpatients were excluded in this study as no confirmed cholera outpatient cases were identified during the data collection period. The patients enrolled in this study were from a high-risk cholera area [8] and all cases were hospitalized, who may have some specific healthcare seeking behavior as the cases considered were serious. This study did not address this issue.

## Conclusions

Our study suggests that by preventing one cholera episode (3 days on an average), we can avert a total cost of 2,278.50 BDT (US$30.40) per household. Simultaneously, the cost of public and not-for-profit private providers can be reduced. This finding has implications regarding policy decisions about investment for cholera prevention programs.

## Competing interests

The authors declare that they have no competing interests.

## Authors’ contributions

ARS, ZI and JAMK designed the study. The study was coordinate by ARS, ZI, IAK, FQ, AS, FC, AIK and JAMK. ARS, ZI and JAMK collected the data. Analysis and interpretation of the data was conducted by ARS, ZI, IAK, FQ, AS, FC, AIK and JAMK. The manuscript was drafted by ARS, ZI, FC and JAMK. All authors critically reviewed and have given final approval of the manuscript.

## Pre-publication history

The pre-publication history for this paper can be accessed here:

http://www.biomedcentral.com/1471-2334/13/518/prepub

## Supplementary Material

Additional file 1 Questionnaire of cost of illness. Click here for file
