# Supplementary material for: Cost of illness for cholera in a high risk urban area in Bangladesh: an analysis from household perspective
Source: BMC Infect Dis. 2013 Nov 4;13:518. doi: 10.1186/1471-2334-13-518 (PMC4228304; doi:10.1186/1471-2334-13-518)
Supplement: Additional file 1 — Questionnaire of cost of illness. [file 1471-2334-13-518-S1.docx]

Introduction of Cholera Vaccine in Bangladesh

International Centre for Diarrhoeal Disease Research, Bangladesh (icddr,b)

Cost of illness due to Cholera:

Number of Ward: _______________ Card Holder______________________01

Name of Area: _________________ Non card Holder and Risk group_____02

Household ID: _________________ Non card Holder and No Risk group_____03

PID Number________________ Receive Oral Cholera Vaccine: Yes____1

Hospital /Patient ID: _________________ No____2

GIS Number: _________________

Age:

Y Y / M M

Gender:

Male _____ 1 Female ________ 2

Admission date: _________________ Time: _________________

Discharge date: _________________ Time: _________________

Name of the patient: _______________________________________

Cost data collection tool from patients

1. **Direct cost**

|  |  | 1^st^ Contact | 2^nd^ Contact | 3rd Contact | 4^th^ Contact |
| --- | --- | --- | --- | --- | --- |
| 101. When you affected with cholera where you get treatment (from first contact to last contact)  Code  01 = Local Pharmacy  02= Local Doctor (MBBS)  03= Dhaka Child Hospital  04= Sowrawardi Hospital  05= SSF Hospital  06= Radda SSF Hospital  07= Al Helal Hospital  08= Modern Hospital  09= Marks E&T Hospital  10= Waida Hospital  11= Dr. Ajmal Hospital  12= Kalshi Child Hospital  13= Mirpur icddrb  14= Mohakhali icddrb  15= Tradition healers  16= Quack  17= Others (please specify) |  | \|  \|  \| \| --- \| --- \| | \|  \|  \| \| --- \| --- \| | \|  \|  \| \| --- \| --- \| | \|  \|  \| \| --- \| --- \| |
| 102. How did you went for treatment purposes  Transport code  1= on foot  2= By –cycle  3= Rickshaw / Van  4= Bus  6= Private car  7= Other ( specify) | Types  of transport  (use code) | \| 1st \|  \|  \| \| --- \| --- \| --- \| \| 2nd \|  \|  \| \| 3rd \|  \|  \| \| 4th \|  \|  \| \| 5th \|  \|  \| \| 6th \|  \|  \| | \| 1st \|  \|  \| \| --- \| --- \| --- \| \| 2nd \|  \|  \| \| 3rd \|  \|  \| \| 4th \|  \|  \| \| 5th \|  \|  \| \| 6th \|  \|  \| | \| 1st \|  \|  \| \| --- \| --- \| --- \| \| 2nd \|  \|  \| \| 3rd \|  \|  \| \| 4th \|  \|  \| \| 5th \|  \|  \| \| 6th \|  \|  \| | \| 1st \|  \|  \| \| --- \| --- \| --- \| \| 2nd \|  \|  \| \| 3rd \|  \|  \| \| 4th \|  \|  \| \| 5th \|  \|  \| \| 6th \|  \|  \| |
| 103a. What time spend for going the treatment centre? | Money | \|  \|  \|  \| \| --- \| --- \| --- \| | \|  \|  \|  \| \| --- \| --- \| --- \| | \|  \|  \|  \| \| --- \| --- \| --- \| | \|  \|  \|  \| \| --- \| --- \| --- \| |
| 103b. Did you spend any money for going the treatment centre? |  | Yes........... 1  No............ 2  Unknown..... 9  Q.105 | Yes........... 1  No............ 2  Unknown..... 9  Q.105 | Yes........... 1  No............ 2  Unknown..... 9  Q.105 | Yes........... 1  No............ 2  Unknown..... 9  Q.105 |
| 104. If yes, what is the amount of money for this purpose? | LiP (BDT) | \|  \|  \|  \|  \| \| --- \| --- \| --- \| --- \| | \|  \|  \|  \|  \| \| --- \| --- \| --- \| --- \| | \|  \|  \|  \|  \| \| --- \| --- \| --- \| --- \| | \|  \|  \|  \|  \| \| --- \| --- \| --- \| --- \| |
| 105. What is the waiting time for receiving this treatment purposes? | Money | \|  \|  \|  \| \| --- \| --- \| --- \| | \|  \|  \|  \| \| --- \| --- \| --- \| | \|  \|  \|  \| \| --- \| --- \| --- \| | \|  \|  \|  \| \| --- \| --- \| --- \| |
| 106 a. Did you spend any money as a registration fee for the particular treatment centre for receiving services? |  | Yes........... 1  No............ 2  Q.107 | Yes........... 1  No............ 2  Q.107 | Yes........... 1  No............ 2  Q.107 | Yes........... 1  No............ 2  Q.107 |
| 106 b. If yes, please specify the amount of money | (BDT) | \|  \|  \|  \|  \| \| --- \| --- \| --- \| --- \| | \|  \|  \|  \|  \| \| --- \| --- \| --- \| --- \| | \|  \|  \|  \|  \| \| --- \| --- \| --- \| --- \| | \|  \|  \|  \|  \| \| --- \| --- \| --- \| --- \| |
| 107 a. Did you spend any money as a bed/cabin rent for accommodation of that centre? |  | Yes........... 1  No............ 2  Q.108 | Yes........... 1  No............ 2  Q.108 | Yes........... 1  No............ 2  Q.108 | Yes........... 1  No............ 2  Q.108 |
| 107 b. If yes, please specify the amount of money | (BDT) | \|  \|  \|  \|  \| \| --- \| --- \| --- \| --- \| | \|  \|  \|  \|  \| \| --- \| --- \| --- \| --- \| | \|  \|  \|  \|  \| \| --- \| --- \| --- \| --- \| | \|  \|  \|  \|  \| \| --- \| --- \| --- \| --- \| |
| 108. Did you spend any money for diagnostic test or other test?  If yes, please specify the amount of money | Stool test  (BDT) | Yes........... 1  No............ 2  Unknown..... 9   \|  \|  \|  \|  \| \| --- \| --- \| --- \| --- \| | Yes........... 1  No............ 2  Unknown..... 9   \|  \|  \|  \|  \| \| --- \| --- \| --- \| --- \| | Yes........... 1  No............ 2  Unknown..... 9   \|  \|  \|  \|  \| \| --- \| --- \| --- \| --- \| | Yes........... 1  No............ 2  Unknown..... 9   \|  \|  \|  \|  \| \| --- \| --- \| --- \| --- \| |
|  | Urine test  (BDT) | Yes........... 1  No............ 2  Unknown..... 9   \|  \|  \|  \|  \| \| --- \| --- \| --- \| --- \| | Yes........... 1  No............ 2  Unknown..... 9   \|  \|  \|  \|  \| \| --- \| --- \| --- \| --- \| | Yes........... 1  No............ 2  Unknown..... 9   \|  \|  \|  \|  \| \| --- \| --- \| --- \| --- \| | Yes........... 1  No............ 2  Unknown..... 9   \|  \|  \|  \|  \| \| --- \| --- \| --- \| --- \| |
|  | Blood test  (BDT) | Yes........... 1  No............ 2  Unknown..... 9   \|  \|  \|  \|  \| \| --- \| --- \| --- \| --- \| | Yes........... 1  No............ 2  Unknown..... 9   \|  \|  \|  \|  \| \| --- \| --- \| --- \| --- \| | Yes........... 1  No............ 2  Unknown..... 9   \|  \|  \|  \|  \| \| --- \| --- \| --- \| --- \| | Yes........... 1  No............ 2  Unknown..... 9   \|  \|  \|  \|  \| \| --- \| --- \| --- \| --- \| |
|  | Other test  (BDT) | Yes.............. 1  No................ 2  Unknown..... 9   \|  \|  \|  \|  \| \| --- \| --- \| --- \| --- \| | Yes............... 1  No............... 2  Unknown..... 9   \|  \|  \|  \|  \| \| --- \| --- \| --- \| --- \| | Yes............... 1  No............... 2  Unknown..... 9   \|  \|  \|  \|  \| \| --- \| --- \| --- \| --- \| | Yes............... 1  No............... 2  Unknown..... 9   \|  \|  \|  \|  \| \| --- \| --- \| --- \| --- \| |
|  | Total  (BDT) | Yes.............. 1  No................ 2  Unknown..... 9   \|  \|  \|  \|  \| \| --- \| --- \| --- \| --- \| | Yes................ 1  No................ 2  Unknown...... 9   \|  \|  \|  \|  \| \| --- \| --- \| --- \| --- \| | Yes............... 1  No............... 2  Unknown..... 9   \|  \|  \|  \|  \| \| --- \| --- \| --- \| --- \| | Yes.............. 1  No............... 2  Unknown..... 9   \|  \|  \|  \|  \| \| --- \| --- \| --- \| --- \| |
| 109 a. Was the service provider come to your house for providing the service? |  | Yes........... 1  No............ 2  Q.110 | Yes........... 1  No............ 2  Q.110 | Yes........... 1  No............ 2  Q.110 | Yes........... 1  No............ 2  Q.110 |
| 109 b. If the service provider come to household then what is the amount of money paid by you as a fee for this service? | (BDT) | \|  \|  \|  \|  \| \| --- \| --- \| --- \| --- \| | \|  \|  \|  \|  \| \| --- \| --- \| --- \| --- \| | \|  \|  \|  \|  \| \| --- \| --- \| --- \| --- \| | \|  \|  \|  \|  \| \| --- \| --- \| --- \| --- \| |
| 110. What is the amount of money that you spend during taking medicine purposes? | (BDT) | \|  \|  \|  \|  \| \| --- \| --- \| --- \| --- \| | \|  \|  \|  \|  \| \| --- \| --- \| --- \| --- \| | \|  \|  \|  \|  \| \| --- \| --- \| --- \| --- \| | \|  \|  \|  \|  \| \| --- \| --- \| --- \| --- \| |
| 111 a. Did you spend any money for buying the following food items like banana, coconut, muri, chira and other? |  | Yes........... 1  No............ 2  Q.112 | Yes........... 1  No............ 2  Q.112 | Yes........... 1  No............ 2  Q.112 | Yes........... 1  No............ 2  Q.112 |
| 111 b . If yes, what is amount of money for this purpose? | (BDT) | \|  \|  \|  \|  \| \| --- \| --- \| --- \| --- \| | \|  \|  \|  \|  \| \| --- \| --- \| --- \| --- \| | \|  \|  \|  \|  \| \| --- \| --- \| --- \| --- \| | \|  \|  \|  \|  \| \| --- \| --- \| --- \| --- \| |
| 112 a. Did you spend any money as tips for your own willingness or against your willingness which consider as a informal payment? |  | Yes........... 1  No............ 2  Q.113 | Yes........... 1  No............ 2  Q.113 | Yes........... 1  No............ 2  Q.113 | Yes........... 1  No............ 2  Q.113 |
| 112 b. If yes, what is amount of money for this purpose? | (BDT) | \|  \|  \|  \|  \| \| --- \| --- \| --- \| --- \| | \|  \|  \|  \|  \| \| --- \| --- \| --- \| --- \| | \|  \|  \|  \|  \| \| --- \| --- \| --- \| --- \| | \|  \|  \|  \|  \| \| --- \| --- \| --- \| --- \| |
| 113 a. Did you bring any person to the treatment centre for helping you based on payment? |  | Yes........... 1  No............ 2  Q.114 | Yes........... 1  No............ 2  Q.114 | Yes........... 1  No............ 2  Q.114 | Yes........... 1  No............ 2  Q.114 |
| 113 b. If yes, what is amount of money for this purpose? | (BDT) | \|  \|  \|  \|  \| \| --- \| --- \| --- \| --- \| | \|  \|  \|  \|  \| \| --- \| --- \| --- \| --- \| | \|  \|  \|  \|  \| \| --- \| --- \| --- \| --- \| | \|  \|  \|  \|  \| \| --- \| --- \| --- \| --- \| |
| 114 a. Did you bought any necessary things like mosquito coil, nets, mug, jar during your stay in treatment centre? |  | Yes........... 1  No............ 2  Q.115 | Yes........... 1  No............ 2  Q.115 | Yes........... 1  No............ 2  Q.115 | Yes........... 1  No............ 2  Q.115 |
| 114 b. If yes, what is amount of money for this purpose? | (BDT) | \|  \|  \|  \|  \| \| --- \| --- \| --- \| --- \| | \|  \|  \|  \|  \| \| --- \| --- \| --- \| --- \| | \|  \|  \|  \|  \| \| --- \| --- \| --- \| --- \| | \|  \|  \|  \|  \| \| --- \| --- \| --- \| --- \| |
| 115. Did you stay outside of your home for taking treatment?  If yes, please specify the amount of hotel rent, food items and other expenditure during that stay? | Hotel or Seat rent  (BDT) | Yes........... 1  No.......... 2  Unknown..... 9   \|  \|  \|  \|  \| \| --- \| --- \| --- \| --- \| | Yes........... 1  No.......... 2  Unknown..... 9   \|  \|  \|  \|  \| \| --- \| --- \| --- \| --- \| | Yes........... 1  No.......... 2  Unknown..... 9   \|  \|  \|  \|  \| \| --- \| --- \| --- \| --- \| | Yes........... 1  No............. 2  Unknown..... 9   \|  \|  \|  \|  \| \| --- \| --- \| --- \| --- \| |
|  | Food items  (BDT) | Yes........... 1  No.......... 2  Unknown..... 9   \|  \|  \|  \|  \| \| --- \| --- \| --- \| --- \| | Yes........... 1  No.......... 2  Unknown..... 9   \|  \|  \|  \|  \| \| --- \| --- \| --- \| --- \| | Yes........... 1  No.......... 2  Unknown..... 9   \|  \|  \|  \|  \| \| --- \| --- \| --- \| --- \| | Yes........... 1  No.......... 2  Unknown..... 9   \|  \|  \|  \|  \| \| --- \| --- \| --- \| --- \| |
|  | Other Expenditure  (BDT) | Yes............. 1  No.............. 2  Unknown..... 9   \|  \|  \|  \|  \| \| --- \| --- \| --- \| --- \| | Yes.......... 1  No.......... 2  Unknown..... 9   \|  \|  \|  \|  \| \| --- \| --- \| --- \| --- \| | Yes........... 1  No.............. 2  Unknown..... 9   \|  \|  \|  \|  \| \| --- \| --- \| --- \| --- \| | Yes........... 1  No............ 2  Unknown..... 9   \|  \|  \|  \|  \| \| --- \| --- \| --- \| --- \| |
|  | Total  (BDT) | Yes........... 1  No.......... 2  Unknown..... 9   \|  \|  \|  \|  \| \| --- \| --- \| --- \| --- \| | Yes........... 1  No.......... 2  Unknown..... 9   \|  \|  \|  \|  \| \| --- \| --- \| --- \| --- \| | Yes........... 1  No.......... 2  Unknown..... 9   \|  \|  \|  \|  \| \| --- \| --- \| --- \| --- \| | Yes........... 1  No.......... 2  Unknown..... 9   \|  \|  \|  \|  \| \| --- \| --- \| --- \| --- \| |
| 116 a. 102. How did you reached your house after taking services?  Transport code  1= on foot  2= By –cycle  3= Rickshaw / Van  4= Bus  6= Private car  7= Other ( specify) | Types  of transport  u( use code) | \| 1st \|  \|  \| \| --- \| --- \| --- \| \| 2nd \|  \|  \| \| 3rd \|  \|  \| \| 4th \|  \|  \| \| 5th \|  \|  \| \| 6th \|  \|  \| | \| 1st \|  \|  \| \| --- \| --- \| --- \| \| 2nd \|  \|  \| \| 3rd \|  \|  \| \| 4th \|  \|  \| \| 5th \|  \|  \| \| 6th \|  \|  \| | \| 1st \|  \|  \| \| --- \| --- \| --- \| \| 2nd \|  \|  \| \| 3rd \|  \|  \| \| 4th \|  \|  \| \| 5th \|  \|  \| \| 6th \|  \|  \| | \| 1st \|  \|  \| \| --- \| --- \| --- \| \| 2nd \|  \|  \| \| 3rd \|  \|  \| \| 4th \|  \|  \| \| 5th \|  \|  \| \| 6th \|  \|  \| |
| 116 b. What is the amount of time for this purpose? | Minutes | \|  \|  \|  \| \| --- \| --- \| --- \| | \|  \|  \|  \| \| --- \| --- \| --- \| | \|  \|  \|  \| \| --- \| --- \| --- \| | \|  \|  \|  \| \| --- \| --- \| --- \| |
| 116 c. What is the amount of money for this purpose? | BDT | Yes......... 1  No.......... 2   \|  \|  \|  \|  \| \| --- \| --- \| --- \| --- \| | Yes..........1  No.......... 2   \|  \|  \|  \|  \| \| --- \| --- \| --- \| --- \| | Yes......... 1  No.......... 2   \|  \|  \|  \|  \| \| --- \| --- \| --- \| --- \| | Yes......... 1  No.......... 2   \|  \|  \|  \|  \| \| --- \| --- \| --- \| --- \| |
| 117 a. After reaching home, did you spend any money for various purpose like medicine, or other that associated with ? |  | Yes.......... 1  No.......... 2  Q.201 | Yes......... 1  No.......... 2  Q.201 | Yes.......... 1  No.......... 2  Q.201 | Yes......... 1  No.......... 2  Q.201 |
| 117 b. If yes, what is amount of money for this purpose? | BDT | \|  \|  \|  \|  \| \| --- \| --- \| --- \| --- \| | \|  \|  \|  \|  \| \| --- \| --- \| --- \| --- \| | \|  \|  \|  \|  \| \| --- \| --- \| --- \| --- \| | \|  \|  \|  \|  \| \| --- \| --- \| --- \| --- \| |
| 118. After meeting the 1^st^ contact of treatment services, how many days ago that you suffered from this disease. | Day | \|  \|  \| \| --- \| --- \| | | | |
| 119. After meeting the last contact of treatment services, how many days suffers of your illness that you think? | Day | \|  \|  \| \| --- \| --- \| | | | |

1. **INDIRECT COSTS**

Now I want to ask you about your occupation and absent of your work for receiving the treatment and other associated aspects.

| 201. How many of your (patients) family member? ( Ascending order of age)  Range of Age   \|  \|  \| \| --- \| --- \|   Under 14 years   \|  \|  \| \| --- \| --- \|   15 to 64 Years   \|  \|  \| \| --- \| --- \|   Above 64 Years | | Total Member | | | \|  \|  \| \| --- \| --- \| | | | | | |  |
| --- | --- | --- | --- | --- | --- | --- | --- | --- | --- | --- | --- | --- | --- | --- | --- | --- | --- | --- | --- |
| 202. What is the educational qualification of the patients?  Class 1 passed ...........................................01  Class 2 passed ...........................................02  Class 3 passed ...........................................03  Class 4 passed ...........................................04  Class 5 passed ...........................................05  Class 6 passed ...........................................06  Class 7 passed ...........................................07  Class 8 passed ...........................................08  Class 9 passed ...........................................09  SSC passed ...............................................10  HSC passed ...............................................12  BA/ B.Com/BSc passed ...........................14 Honors passed .............................. ............16 Masters and higher passed.........................17  No education .............................................66  Other (specify) .........................................77  N/A …………............................................88 | \|  \|  \| \| --- \| --- \|   Code    N/A --------------------------------🡪Q.209 | | | | | | | | | | |
| 203. What is the occupation of Patient?  ( Occupation code)  Looking for a job ......................................01  Housewife................................................02  Beggar........................................................03  Pensioner ...................................................04  Home service/ Servant...............................05  Motor Driver ............................................06  Rickshaw/van Driver................................07  Day labor...................................................08  Fisherman ................................................09  Tailor/ Berber ..........................................10  Business ..................................................11  Services.....................................................12  Teacher......................................................13  Doctor ......................................................14  Engineer....................................................15  Internship..................................................16  Student......................................................17  Hawker.....................................................18  Germen’s labor.........................................19  Benaroshis’s labor ...................................20  Other (specify)....................,.................... 77  Unknown ........................................,..... 99  N/A... ……………….……………….….88 | \|  \|  \| \| --- \| --- \|   Primary occupation   \|  \|  \| \| --- \| --- \|   Secondary occupation | | | | | | | | | | |
| 204. Monthly income of patients | Yes............... 1   \|  \|  \|  \|  \| \| --- \| --- \| --- \| --- \|   No................. 2  N/A ....... 88  Q.209 | | | | | | | | | | |
| 205. When the person affected with cholera during that time, did he/she engaged a paying job? | Yes ..........................................1  No ……..…………….……..2 | | | | | | | | | | |
| 206. How many days he/she absent from work / school/ institution | Day | | | \|  \|  \|  \| \| --- \| --- \| --- \| | | | | | | | |
| 207. Did you make any income loss due to this absent from work? | Yes ........................................................01  No ……..……………………....……..02  Unwillingness to answer...................... 03  Q.209 | | | | | | | | | | |
| 208. If Yes, please specify the amount of money | \|  \|  \|  \|  \| \| --- \| --- \| --- \| --- \|   BDT | | | | | | | | | | |
| 209. What monthly income of your family? | \|  \|  \|  \|  \| \| --- \| --- \| --- \| --- \|     BDT | | | | | | | | | | |
| 210. During illness, did anybody taking care of the patients? If yes, please specify the following information. In case of educational qualification use the previous educational code | Attendant 1 | | | | | \|  \|  \| \| --- \| --- \|   ‡ckv Educational qualification | | | \| Day \|  \|  \| \| --- \| --- \| --- \| \| Hour \|  \|  \| | |  |
|  | Attendant 2 | | | | | \|  \|  \| \| --- \| --- \|   ‡ckv Educational qualification | | | \| Day \|  \|  \| \| --- \| --- \| --- \| \| Hour \|  \|  \| | |  |
|  | Attendant 3 | | | | | \|  \|  \| \| --- \| --- \|   ‡ckv Educational qualification | | \| Day \|  \|  \| \| --- \| --- \| --- \| \| Hour \|  \|  \| | | |  |
| 211. Please specify the occupation and monthly income of the attendant ( use the previous occupational code ) | Attendant 1 | | | | | \|  \|  \| \| --- \| --- \|   ‡ckv Occupational code | | \|  \|  \|  \|  \| \| --- \| --- \| --- \| --- \|   monthly income | | |  |
|  | Attendant 2 | | | | | \|  \|  \| \| --- \| --- \|   ‡ckv Occupational code | | \|  \|  \|  \|  \| \| --- \| --- \| --- \| --- \|   monthly income | | |  |
|  | Attendant 3 | | | | | \|  \|  \| \| --- \| --- \|   ‡ckv Occupational code | | \|  \|  \|  \|  \| \| --- \| --- \| --- \| --- \|   monthly income | | |  |
| 212 a. Did the attendant faces any income losses due to caring the patients? | Yes…............. 1  No................. 2  N/A………... 88  Q.213 | | | | | | | | | |  |
| 212 b. If yes, what is amount of money for this purpose? | \|  \|  \|  \|  \| \| --- \| --- \| --- \| --- \|   BDT | | | | | | | | | |  |
| 213. For this purpose, did attendants spend any money during that time?  If yes, please specify the amount of hotel rent, food items and other expenditure during that stay? |  | | 1^st^ Contact | | | 2^nd^ Contact | 3rd Contact | | | 4^th^ Contact |  |
|  | Hotel or Seat rent  (BDT) | | \|  \|  \|  \|  \| \| --- \| --- \| --- \| --- \| | | | \|  \|  \|  \|  \| \| --- \| --- \| --- \| --- \| | \|  \|  \|  \|  \| \| --- \| --- \| --- \| --- \| | | | \|  \|  \|  \|  \| \| --- \| --- \| --- \| --- \| |  |
|  | Food items  (BDT) | | \|  \|  \|  \|  \| \| --- \| --- \| --- \| --- \| | | | \|  \|  \|  \|  \| \| --- \| --- \| --- \| --- \| | \|  \|  \|  \|  \| \| --- \| --- \| --- \| --- \| | | | \|  \|  \|  \|  \| \| --- \| --- \| --- \| --- \| |  |
|  | Transport  (BDT) | | \|  \|  \|  \|  \| \| --- \| --- \| --- \| --- \| | | | \|  \|  \|  \|  \| \| --- \| --- \| --- \| --- \| | \|  \|  \|  \|  \| \| --- \| --- \| --- \| --- \| | | | \|  \|  \|  \|  \| \| --- \| --- \| --- \| --- \| |  |
|  | Others  (BDT) | | \|  \|  \|  \|  \| \| --- \| --- \| --- \| --- \| | | | \|  \|  \|  \|  \| \| --- \| --- \| --- \| --- \| | \|  \|  \|  \|  \| \| --- \| --- \| --- \| --- \| | | | \|  \|  \|  \|  \| \| --- \| --- \| --- \| --- \| |  |
|  | Total  (BDT) | | \|  \|  \|  \|  \| \| --- \| --- \| --- \| --- \| | | | \|  \|  \|  \|  \| \| --- \| --- \| --- \| --- \| | \|  \|  \|  \|  \| \| --- \| --- \| --- \| --- \| | | | \|  \|  \|  \|  \| \| --- \| --- \| --- \| --- \| |  |

**Thank you for your cooperation**

Name of the field Investigator: Name of the field Supervisor

Signature: _____________ Signature______________

Date:_______________ Date: _________________
